# A Tröger’s Base-Derived Covalent Organic Polymer Containing Carbazole Units as a High-Performance Supercapacitor

**DOI:** 10.3390/polym13091385

**Published:** 2021-04-24

**Authors:** Ahmed F. M. EL-Mahdy, Johann Lüder, Mohammed G. Kotp, Shiao-Wei Kuo

**Affiliations:** Department of Materials and Optoelectronic Science, National Sun Yat-Sen University, Kaohsiung 80424, Taiwan; Johann.lueder@mail.nsysu.edu.tw (J.L.); mgmkotp@yahoo.com (M.G.K.); kuosw@faculty.nsysu.edu.tw (S.-W.K.)

**Keywords:** carbazole, Tröger’s base, conjugated microporous polymer, regioselectivity, supercapacitors

## Abstract

Porous organic polymers have been received considerable attention due to their heteroatom-containing structures and high surface areas, which can offer high electrochemical performance in energy applications. The majority of reported Tröger’s base-functionalized porous organic polymers have been applied as effective candidates for sensing and gas separation/adsorption, while their use as electrode materials in supercapacitors is rare. Here, a novel covalent microporous organic polymer containing carbazole and Tröger’s base CzT-CMOP has been successfully synthesized through the one-pot polycondensation of 9-(4-aminophenyl)-carbazole-3,6-diamine (Cz-3NH_2_) with dimethoxymethane. The polycondensation reaction’s regioselectivity was studied using spectroscopic analyses and electronic structure calculations that confirmed the polycondensation occurred through the second and seventh positions of the carbazole unit rather than the fourth and fifth positions confirmed by first-principles calculations. Our CzT-CMOP exhibited high thermal stability of approximately 463.5 °C and a relatively high Brunauer–Emmett–Teller surface area of 615 m^2^ g^−1^ with a nonlocal density functional theory’s pore size and volume of 0.48 cm^3^ g^−1^ and 1.66 nm, respectively. In addition, the synthesized CzT-CMOP displayed redox activity due to the existence of a redox-active carbazole in the polymer skeleton. CzT-CMOP revealed high electrochemical performance when used as active-electrode material in a three-electrode supercapacitor with an aqueous electrolyte of 6 M KOH, and it showed specific capacitance of 240 F g^−1^ at a current density of 0.5 A g^−1^ with excellent stability after 2000 cycles of 97% capacitance retention. Accordingly, such porous organic polymer appears to have a variety of uses in energy-related applications.

## 1. Introduction

Since the industrial revolution, economic and industrial development relied on the tremendous use of fossil fuels such as coal, gas, and mineral oil [1,2]. For more efficient use of the converted energy, renewable fuel technologies are now often combined with some form of energy storage technologies such as batteries and supercapacitors, which can mitigate and reduce harmful impacts on global warming, toxic atmospheres, and inadequate habitats [3,4]. Emerging mobile electronic devices and electric mobility energy storage devices have an important role in our daily life; however, many technical challenges remain unsolved for them, such as safety and sustainability, besides increasing demand for higher performance devices. Therefore, there has been great interest in refining and developing more efficient energy storage devices [5]: electrochemical energy storage, including batteries and supercapacitors (SCs). Supercapacitors feature a high surface area of the electrode materials and thin electrolytic dielectrics to achieve capacitance values greater than conventional capacitors. As compared to batteries, SCs have some disadvantages, including a quick discharge, low energy density, and non-constant voltage output [6,7]. However, SCs are, in general, not more expensive than batteries and have other advantages over batteries such as high volumetric capacitance, long cycle life, good operational safety, low internal resistance, wide working temperature ranges, flexible packaging, and small size, making them the preferred choice in industrial applications [8,9,10,11]. These features are attributed to the supercapacitor mechanism for energy storage. SCs can be divided into three classes that are: electrical double-layer capacitors (EDLC), pseudocapacitors, and hybrid capacitors [12,13,14]. Because the supercapacitor performance is a materials-related optimization challenge, many carbon-based materials, including carbon nanotubes, activated carbons, graphenes, and graphene-aerogels, have developed [15,16,17,18]. However, these carbon materials suffer from pore wetting by electrolyte ions, which can lower a supercapacitor’s performance [19,20]. As a result, the development of new electrode materials for outstanding supercapacitor performance is a hot research field.

Compared to traditional inorganic (silica, zeolite) and carbon porous materials, microporous organic polymers (POPs) have gained tremendous interest due to their unique properties such as permanent porosity, variable morphologies, high surface area, low density, easy functional modification, various synthetic strategies, and excellent thermal and chemical stability [21,22,23]. Recently, novel POPs have been reported, including covalent triazine frameworks (CTFs), covalent organic frameworks (COFs), polymers of intrinsic microporosity (PIMs), hyper-cross-linked polymers (HCPs), conjugated microporous polymers (CMPs), and porous aromatic frameworks (PAFs) [24,25,26,27,28,29,30,31,32,33,34,35,36]. These unique features of POPs encouraged the use of POPs in several technological applications such as sensing, luminescence, catalysis, gas adsorption, energy storage, water splitting, and semiconducting devices [37,38,39,40,41,42,43,44,45]. Much research work has been expended in the applications of CMPs in energy storage domains [46,47]. For supercapacitors, CMPs are generally used as carbon, nitrogen, and heteroatom sources for the preparation of non-doped and doped microporous carbon electrodes by pyrolysis process. The presence of dopants such as nitrogen atoms and other heteroatoms in the resulting carbons strongly enhance the supercapacitor’s capacitances via increasing their conductivities [48,49,50,51]. Regrettably, the pyrolyzed carbons from such polymers have either poor capacitance or restricted cycling durability, with little discernible advantage over traditional porous carbon materials. Recently, the use of CMPs without a pyrolysis process has been developed [48]. Incorporating redox-active moieties such as triphenylamine, porphyrin, diamino-anthraquinone, and pyridine into the skeleton of porous polymers is a good way to improve their capacitance [27,48,52,53]. However, the capacitance of such polymers remains relatively low due to their poor inherent conductivity. Therefore, the development of new conjugated micropores polymers having redox-active moiety and high inherent conductivity is strongly need.

**Scheme 1:**
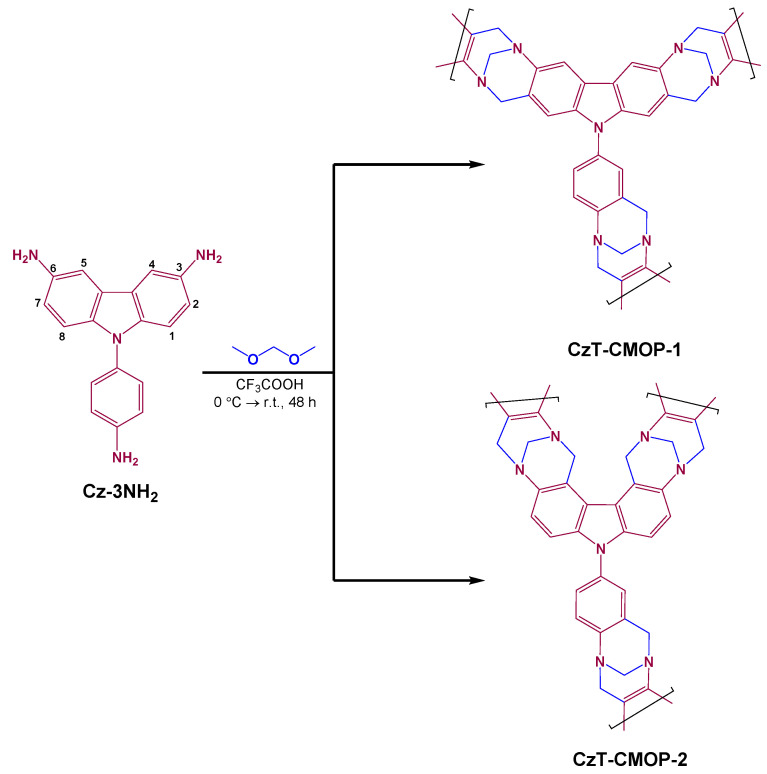
Synthesis of the covalent organic Tröger’s base polymer CzT-CMOP.

Very recently, POPs containing Tröger’s base (TB) moieties have attracted significant interest because of the V-shaped geometry, rigidity, and *C*_2_ symmetry of the Tröger’s base [54,55,56,57]. In addition, the TB-derived POPs can be easily prepared by the metal-free condensation reaction of aromatic amines with dimethoxymethane at ambient temperature [55,56,57,58]. To date, several TB-derived POPs have been constructed using amino-functionalized pyrene, triphenyltriazine, triphenylbenzene, and tetraphenylethene and then were used as efficient materials for sensing catalysis, gas separation, and storage [55,56,57,58]. To the best of our knowledge, the use of TB-derived microporous organic polymer for the preparation of carbon electrode material for supercapacitor has been once reported by the carbonization of TB-derived polymer at 1100 °C [59]. The preparation of TB-derived polymer containing electrochemical redox-active moiety for energy storage application has not been reported. Therefore, in this study, we demonstrated an efficient strategy to prepare a covalent organic polymer containing carbazole and Tröger’s base (CzT-CMOP, Scheme 1) through the one-pot polycondensation of a rigid building linker named 9-(4-aminophenyl)-carbazole-3,6-diamine with dimethoxymethane at ambient temperature. The regioselectivity of the polycondensation reaction is discussed using nuclear magnetic resonance analyses and electronic structure calculations. The resulting polymer was then tested as a potential electrode for supercapacitor and its capacitive including cyclic voltammetry, galvanostatic charge/discharge, and cyclic stability were studied.

## 2. Materials and Methods

### 2.1. Materials

Reagents and solvents were obtained from commercial sources and used as received. Carbazole (95%), acetic anhydride (99%), copper (II) nitrate trihydrate (98%), and acetic acid (99.8%) were obtained from Sigma-Aldrich. Dimethoxymethane (95%) and palladium on activated carbon, 10% Pd/C, were purchased from Acros. 1-Fluoro-4-nitrobenzene (99%), potassium carbonate (99%), potassium hydroxide (KOH, 90%), trifluoromethanesulfonic acid (F_3_CSO_3_H, 98%), and hydrazine hydrate (98%) were ordered from Alfa Aesar. Methanol (99.8%), acetone (99.5%), ethylacetate (EA, 99.8%), petroleum ether (≥90%), tetrahydrofuran (THF, 99.9%), ethanol (99.8%), and dichloromethane (DCM, 99.8%) were obtained from Showa (Tokyo, Japan).

### 2.2. Synthesis of 9-(4-Aminophenyl)-Carbazole-3,6-Diamine (Cz-3NH_2_)

Cz-3NH_2_ was synthesized according to our previous study, with slight modification [27]. In a 250-mL two-neck round-bottomed flask, 3,6-dinitro-9-(4-nitrophenyl)-carbazole (2.0 g, 5.30 mmol) and 10% Pd/C (0.14 g) were suspended in a cosolvent of 1,4-dioxane and ethanol (2:1, 60 mL). Then, hydrazine monohydrate (6.4 mL) was dropwise added. The suspension solution was heated under inert atmosphere at 100 °C for 2 days. After the starting materials were consumed (TLC), the reaction mixture was filtered, cooled, and concentrated with rotatory evaporation and then poured on cold distilled water (100 mL) to provide a solid product. The resulting product was isolated by vacuum filtration. The desired Cz-3NH_2_ was obtained as pale green crystals (1 g, 70%), m.p.: 126–128 °C (DSC). FT-IR (powder): 3403, 3340, 3211, 3039, 1618, 1519, 1491, 1468, 1340, 1315, 1283, 1215, 837, 810. ^1^H NMR (DMSO-*d_6_*, 25 °C, 500 MHz): δ = 7.11 (s, 2H), 7.08 (d, *J =* 8.5 Hz, 2H), 6.96 (d, *J =* 8.5 Hz, 2H), 6.75 (d, *J =* 8.5 Hz, 2H), 6.68 (d, *J =* 8.5 Hz, 2H) (Figure S1). ^13^C NMR ((DMSO-*d_6_*, 25 °C, 125 MHz): δ = 147.45, 141.22, 134.49, 126.95, 126.98, 122.74, 114.81, 114.60, 109.64, 103.72 (Figure S2).

### 2.3. Carbazole- and Tröger’s Base-Derived Covalent Microporous Organic Polymer

In a 50-mL two-neck round-bottomed flask under argon atmosphere, Cz-3NH_2_ (288 mg, 1 mmol) was slowly added to a cold trifluoroacetic acid (20 mL) in an ice bath. After 15 min, a solution of dimethoxymethane (1 mL, 11.4 mmol) in iced-trifluoroacetic acid (5 mL) was dropped. The resulting reaction mixture allowed to stir at 25 °C for 2 days. Then the obtained solution was poured into cooled water (50 mL), and then ammonium solution (20% in water) was dropped until pH = 10 was reached. After stirring for 3 h, the crude carbazole- and Tröger’s base-derived polymer was isolated and washed with water, ethanol, and acetone. Then, the desired polymer then underwent to purify with Soxhlet extraction using methanol for 24 h and hexane for 24 h. The purified polymer was kept at 100 °C for 1 day to produce a brown solid (85%).

### 2.4. Electrochemical Measurements

Working Electrode Cleaning: The glass carbon electrode (GCE) was polished several times prior to use with 0.05 μm alumina powder, washed with ethanol after each polishing process, sonically cleaned for several minutes in a water bath, washed with ethanol, and then air-dried.

Electrochemical Characterization: The electrochemical measurements were carried out using a three-electrode cell in an Autolab potentiostat (PGSTAT204), and an aqueous solution of KOH (6 M) was used as an electrolyte. The GCE, Pt wire, and Hg/HgO (RE-61AP, BAS) were used as working (diameter: 5.61 mm; 0.2475 cm^2^), counter, and reference electrodes, respectively. A slurry of carbazole- and Tröger’s base-derived covalent microporous organic polymer was prepared by mixing the active polymer material (80 wt.%) with Nafion solution (10 wt.%), and carbon black (10 wt.%) in ethanol (2 mL) and then sonicating for 1 h. A portion of this slurry (10 µL) was pipetted onto the tip of the glass carbon electrode and dried in air for 30 min prior to use. The electrochemical performance was studied through cyclic voltammetry by applying different sweep rates up to 200 mV s^−1^ and through the galvanostatic charge/discharge method by applying a potential range from 0.6 to –1.0 V at various current densities up to 20 A g^−1^. The galvanostatic charge/discharge curves of polymer were used to calculate the specific capacitance of polymer through the following equation:

*C*_s_ = (*I*∆*t*)/(*m*∆*V*) (1)

where *C*_s_ (F g^−1^) is the specific capacitance of the supercapacitor, *I* (A) is the discharge current, Δ*V* (V) is the potential window, Δ*t* (s) is the discharge time, and *m* (g) is the total mass of the polymer on the electrode.

### 2.5. Density Functional Theory Calculations

The atomic and electronic structure of the fully relaxed monomer units of Cz-NH3, CzT-CMOS-1, and CzT-CMOS-2 were studied using density functional theory (DFT) employing the Becke-3-parameter- Lee−Yang−Parr (B3LYP) hybrid functional and the 6-31G(d) basis to describe exchange-correlation effects and represent electronic states, respectively [60,61,62,63,64].

## 3. Results and Discussion

### 3.1. Synthesis and Electronic Structure Calculations of CzT-CMOP

**Figure 1:**
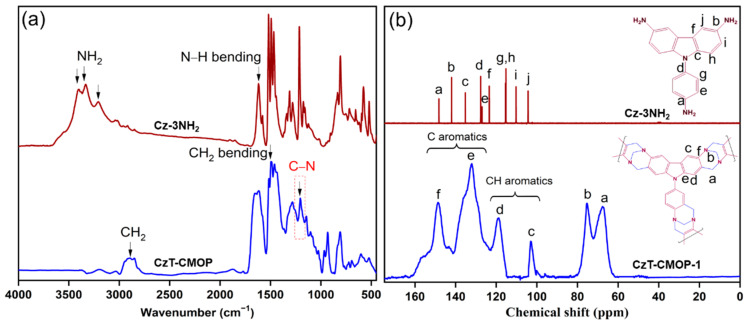
(**a**) FTIR of Cz-3NH_2_ (wine color) and CzT-CMOP (blue color). (**b**) ^13^C NMR spectrum of Car-3NH_2_ (wine color) and solid-state ^13^C CP/MAS NMR spectrum of CzT-CMOP.

One of this study’s main objectives is the evaluation of the activity and regioselectivity of the Tröger’s base polymerization toward 9-(4-aminophenyl)-carbazole-3,6-diamine (Cz-3NH_2_). On treatment of Cz-3NH_2_ with dimethoxymethane in an excess of trifluoroacetic acid at room temperature for 48 h under nitrogen atmosphere, the polymerization may proceed to produce carbazole- and Tröger’s base-derived covalent microporous organic polymer (CzT-CMOP-1) or the other isomeric polymer (CzT-CMOP-2) (Scheme 1). CzT-CMOP-1 can be produced, when the polymerization is carried out on the second and seventh positions of the carbazole unit, while CzT-CMOP-2 can be produced, when the polymerization occurred on the fourth and fifth positions of the carbazole unit. The resulting CzT-CMOP was isolated as a brown solid and was found to be insoluble in common organic solvents, including acetone, dimethyl sulfoxide, tetrahydrofuran, dimethylformamide, methanol, and dioxane. We used Fourier transform infrared (FTIR) spectroscopy to investigate the formation of CzT-CMOP and to determine its functional groups. Figure 1a showed the comparison of FTIR spectra of the formed CzT-CMOP and the starring monomer Cz-3NH_2_. The distinctive vibration bands in the range from 3402 to 3210 cm^−1^ centered on 1619 cm^−1^ in the FTIR spectrum of Cz-3NH_2_, which attributed to the N–H symmetric stretches and N–H symmetric bend, respectively, were absent, indicating the incorporation of Cz-3NH_2_ into the carbazole- and Tröger’s base-derived covalent microporous organic polymer. In addition, the presence of new vibration bands in the range 2984–2784 cm^−1^ for the CH_2_ symmetric stretches and a new vibration band at 1197 cm^−1^ for the C–N symmetric stretch confirmed the formation of CMOP. However, due to the structural similarity between the isomers, these FTIR data cannot distinguish which of those isomers, i.e., CzT-CMOP-1 or CzT-CMOP-2, is produced, and additional insights are needed.

The regioselectivity of Tröger’s base polymerization and the formation of CzT-CMOP-1 rather than CzT-CMOP-2 were investigated using solid-state ^13^C CP/MAS NMR spectroscopy and electronic structure calculations. Figure 1b showed the ^13^C NMR spectrum of the monomer Cz-3NH_2_ in DMSO-*d_6_* and solid-state ^13^C CP/MAS NMR spectrum of the resulting CMOP. Our synthesized CMOP revealed characteristic vibrations at 75.32 and 67.67 ppm, which can be assigned to the methylene (CH_2_) carbons, indicating the successful formation of the Tröger’s base unit. The other characteristic vibrations were at 148.77, 131.91, 118.81, and 102.87 ppm and assigned to aromatic (C, and C–H) carbons. The ^13^C CP/MAS NMR data of CMOP are approximately consistent with the ^13^C NMR of the monomer Cz-3NH_2_. The disappearance of the aromatic (C_i_) carbons in the second and seventh positions of the Cz-3NH_2_ after Tröger’s base polymerization, in addition to the existence of the aromatic (C_j_) carbons in the fourth and fifth positions, confirms that the chemical structure of the resulting CMOP is in agreement with the product CzT-CMOP-1. This observation is further supported by electronic structure calculations.

**Figure 2:**
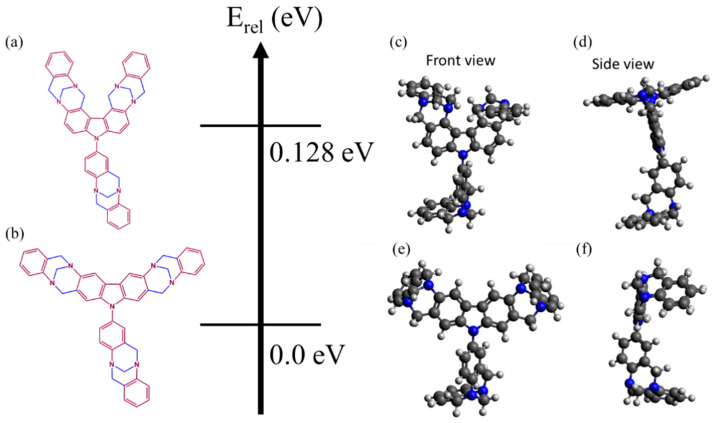
Chemical (left) and DFT-optimized structures (right) of CzT-CMOP-1 (**a**,**c**,**e**) and CzT-CMOP-2 (**b**,**d**,**f**), respectively, arranged next to the relative total energy scale.

The different isomers’ optimized structures are shown in Figure 2, together with their relative total energies. The most likely isomeric form (i.e., CzT-CMOP-1 or CzT-CMOP-2 shown in Figure 2a,b) of the monomer units can be determined from the difference of the former quantity. According to the B3LYP structure optimization, a CzT-CMOP-1 monomer is about 128 m eV more stable than the one of CzT-CMOP-2. While a comprehensive analysis of the reaction mechanism, including transition state, rate constants, etc., is beyond the scope of this study, the total energy difference between the two isomers can provide some insight into which isomer structure is more likely to be in our experiment.

### 3.2. TGA, FE-SEM, TEM, and BET Analyses of CzT-CMOP

**Figure 3:**
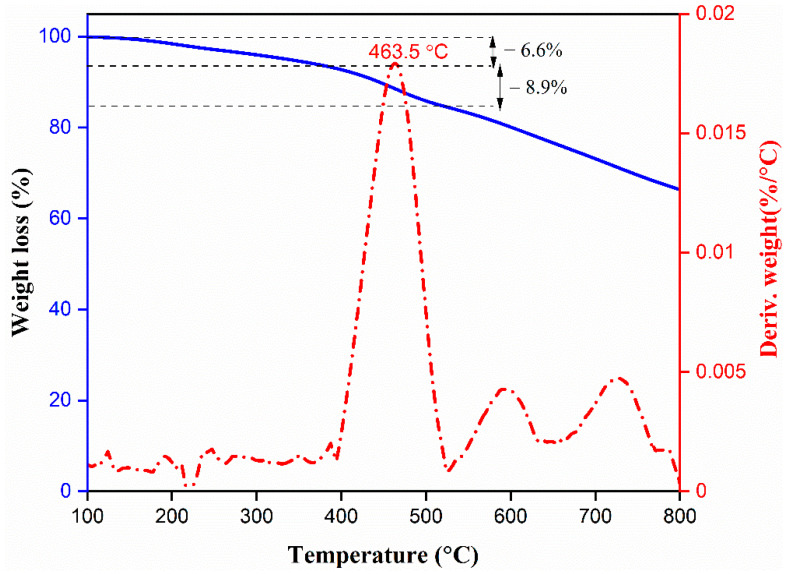
Thermal stability of the CzT-CMOP-1.

The thermal stability of porous polymers is one of the main criteria for their potential uses in commercial energy storage and electrochemical supercapacitor technology. Therefore, we studied the thermal stability of our synthesized carbazole- and Tröger’s base-derived covalent microporous organic polymer by recording the thermal gravimetric analysis (TGA) in a temperature range from 40 to 800 °C under nitrogen atmosphere with a heating rate of 10 °C min^−1^. As shown in Figure 3, the CzT-CMOP-1 exhibited an early weight loss of around 6.6% before 387 °C, which can be attributed to the desorption of the captured solvents [65,66]. In addition, CzT-CMOP-1 began to decompose at a temperature of 463.5 °C. After heating to 800 °C, the weight loss of CzT-CMOP-1 was approximately 44% of the original weight. The differential scanning calorimetry analysis of our CzT-CMOP-1 did not show any phase transition (Figure S3).

**Figure 4:**
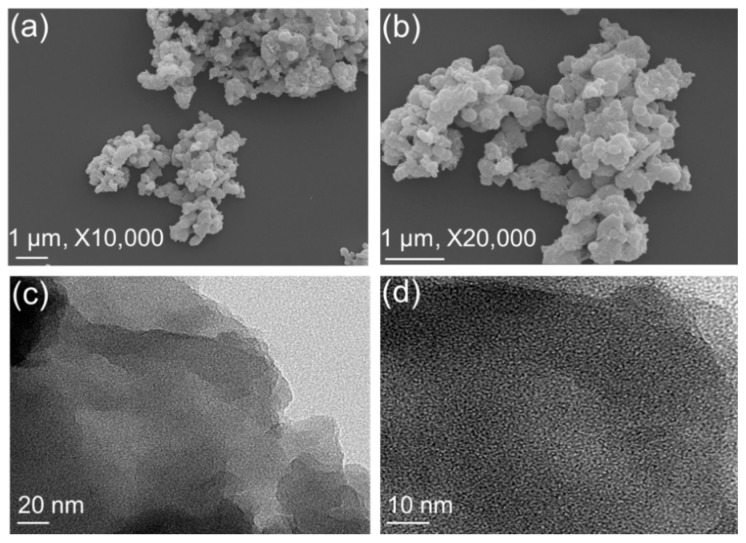
(**a**,**b**) FE-SEM and (**c**,**d**) TEM images of the CzT-CMOP-1, recorded at various magnifications.

**Figure 5:**
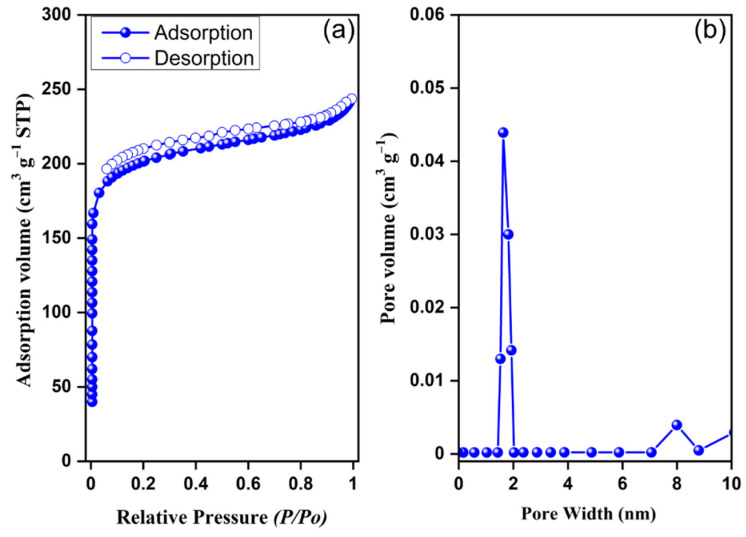
(**a**) Nitrogen adsorption (●) and desorption (○) isotherm and (**b**) pore size distribution profile of the CzT-CMOP.

The morphology and surface crystallinity of our carbazole- and Tröger’s base-derived covalent microporous organic polymer were investigated using field emission scanning electron microscopy (FE-SEM) and transmission electron microscopy (TEM) (Figure 4). The FE-SEM images of CzT-CMOP-1 revealed an irregular spherical morphology with a dimension of several micrometers, which could be caused by the accumulation of nanoscale polymer particles (Figure 4a,b). On the other hand, the TEM images of CzT-CMOP-1 observed π-π stacked layers on the particle edges of our synthesized polymer (Figure 4c,d). The porous nature of our carbazole- and Tröger’s base-derived covalent microporous organic polymer was estimated by recording the nitrogen sorption measurement at 77 K. As presented in Figure 5a, CzT-CMOP-1 has characteristic nitrogen sorption of the type I isotherm, which featured with sharp nitrogen adsorption in the relative pressure range (*P*/*P_0_*) lower than 0.1 bar and unremarkable nitrogen adsorption in a relative pressure higher than 0.1 bar. The absence of a hysteresis loop in the resultant isotherm indicated the microporous nature of our synthesized CzT-CMOP-1. The Brunauer–Emmett–Teller (BET) specific surface area of CzT-CMOP-1 was evaluated from its sorption curve to be 615 m^2^ g^−1^. The pore volume of CzT-CMOP-1 was found to be 0.48 cm^3^ g^−1^. Figure 5b presented the pore size distribution of the polymer, which can be estimated by the classic nonlocal density functional theory (NL-DFT). The CzT-CMOP-1 exhibited a pore size of 1.66 nm.

### 3.3. Supercapacitor Application

**Figure 6:**
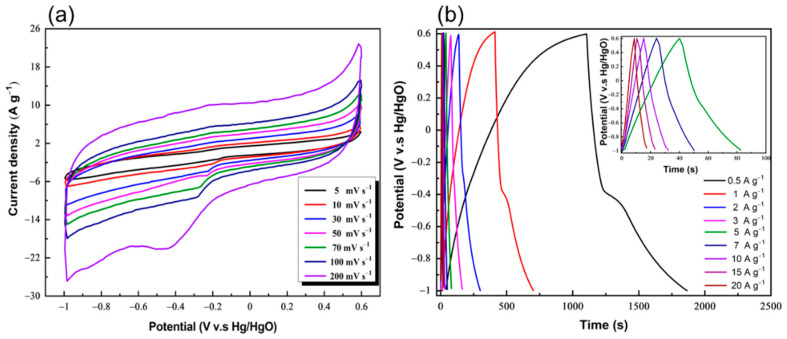
(**a**) Cyclic voltammograms of the CzT-CMOP-1 at different scan rates (mV s^−1^) in 6 M KOH and (**b**) galvanostatic charge-discharge curves of the CzT-CMOP-1 at different current densities (A g^−1^).

The high surface area and orderly pore size electrodes are the primary prerequisites for outstanding electrode efficiency in an electrical double-layer capacitor (EDLC) based supercapacitor. In addition, it has been recently reported that the porous organic polymers received significant interest as electrode materials due to their ability to incorporate redox-active moieties in their networks [27]. Therefore, we synthesized a new conjugated microporous organic polymer containing the redox-active carbazole moiety and the rich-nitrogen Tröger’s base in its pore skeleton, in addition to its high specific surface area and uniform pore size. Then, we examined the formed conjugated microporous organic polymer as an electrode in the electrochemical supercapacitor. The electrochemical characteristics of the CzT-CMOP-1 were estimated using cyclic voltammetry (CV), galvanostatic charge/discharge (GCD), and cyclic stability measurements in a three-electrode mode and in the presence of a basic aqueous electrolyte of KOH (6 M). The cyclic voltammetry of our carbazole- and Tröger’s base-derived covalent microporous organic polymer was obtained by varying the potential from 0.6 to −1.0 V at sweep rates of 5, 10, 30, 50, 70, 100, and 200 mV s^−1^. The Hg/HgO electrode was used as a reference electrode. As shown in Figure 6a, the CzT-CMOP-1 observed nearly rectangle-like CV curves, confirmed that the capacitive response of our CzT-CMOP-1 electrode arisen from EDLC. It has been reported that the EDLC is mainly arised due to the formation of electrostatic double layers at the interface between the electrolyte and electrode [18]. Therefore, the EDLC behavior of CzT-CMOP-1 can be attributed to the formation of electrostatic interfaces between the electrolyte and CzT-CMOP-1 layers. Additionally, the presence of nitrogen atoms in the CzT-CMOP-1 skeleton is strongly increased the interlayer distances between the polymer layers, leading to enhance ion-diffusion and electron transfer into the CzT-CMOP-1 layers [67]. This behavior is expected to produce a supercapacitor electrode of CzT-CMOP-1 with high capacitance performance. It is important to observe that the rectangle-like shape of CV curves was retained at all sweep rates, even at the highest 200 mV s^−1^, indicating excellent charge propagation across the CzT-CMOP-1 electrode [68]. In addition, the CV curves of our polymer exhibited small humps, suggesting a combination of a minor pseudocapacitor (PC) and a major EDLC. These humps were generated due to the Faradic redox currents, which could stem from the presence of redox-active carbazole in the CzT-CMOP-1 framework. Furthermore, the capacitive properties of our CzT-CMOP-1 electrode were confirmed by GCD measurements over different current densities of 0.5, 1, 2, 3, 5, 7, 10, 15, and 20 A g^−1^ (Figure 6b).

**Figure 7:**
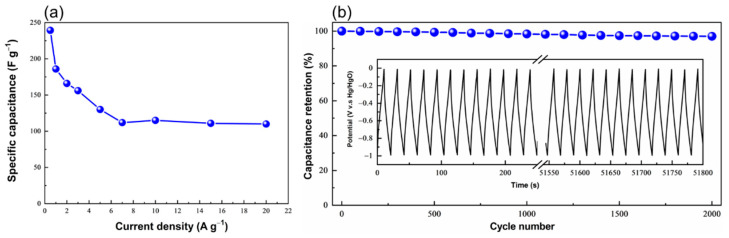
(**a**) Specific capacitances of the CzT-CMOP-1 at different current densities (A g^−1^), and (**b**) cycling performance of the CzT-CMOP-1 recorded at a density of 10 A g^−1^ for 2000 cycles.

**Figure 8:**
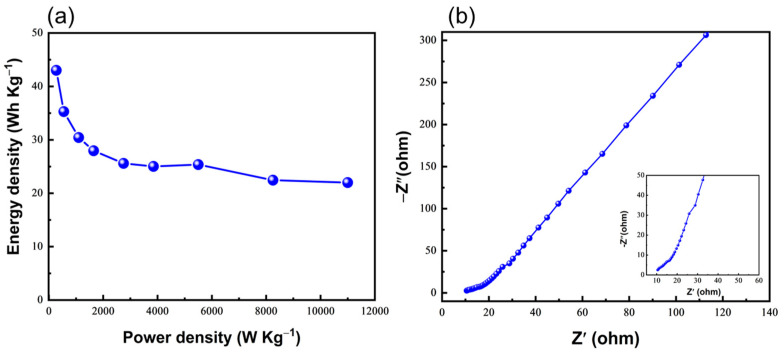
(**a**) Ragone and (**b**) Nyquist plots for CzT-CMOP-1 electrode in 6 M KOH.

In agreement with CV data, the CzT-CMOP-1 electrode exhibited triangular charge-discharge curves featuring slight bend, which showed both EDLC and pseudocapacity behaviors generated from Faradic electrochemical redox reactions [69,70]. Figure 7a presented the specific capacitances of our CzT-CMOP-1 electrode, which is estimated from its GCD curves, by applying Equation (S1). The achieved specific capacitance value for the CzT-CMOP-1 reached 240 F g^−1^ at a current density of 0.5 A g^−1^. Particularly, the CzT-CMOP-1 electrode demonstrated capacitance retention of 46% of its low-current capacitance upon increasing the current density to 20 A g^−1^ (Figure 7a). The cyclic life of the CzT-CMOP-1 electrode was examined by using 2000 charge-discharge cycles at a constant current density of 10 A g^−1^. As shown in Figure 7b, the CzT-CMOP-1 electrode observed efficient stability of 97% capacitance retention. The Ragone plot of our polymer revealed a high energy density of the CzT-CMOP-1 of 43 Wh kg^−1^ (Figure 8a). Electrochemical impendence spectroscopy (EIS) was used to further investigate the electrochemical properties (conductivity and charge transfer) of the CzT-CMOP-1 electrode over an amplitude of 5 mV and a frequency range of 0.01 Hz-100 KHz. As shown in Figure 8b, the Nyquist plot of the CzT-CMOP-1 electrode featured an oblique line in the low-frequency field, suggesting the extreme capacitive behavior of CzT-CMOP-1 and a semicycle in the high-frequency field, demonstrating strong pore conductivity for the electrolyte ions [71]. By investigating the intercept of the Z’ axis in the high-frequency field, we were able to calculate the intrinsic ohmic resistance (Rs), which reflected the conductivity of the CzT-CMOP-1 electrode. The polymer revealed an Rs value of 9.8 Ω (Figure 8a, insert), indicating the high conductivity of the CzT-CMOP-1 electrode. In comparison, the electrochemical specific capacitance of the CzT-CMOP-1 electrode exceeded or, at least, is comparable to the reported ones of porous organic polymers (Table S1). Therefore, we believe that the newly synthesized carbazole- and Tröger’s base-derived covalent microporous organic polymer will open the door to use Tröger’s base-derived polymers as promising electrodes for energy storage applications. Additionally, the chemical stability of our CzT-CMOP-1 in the measurement electrolyte was investigated by submerging the CzT-CMOP-1 in an aqueous solution of KOH (6 M). After 48 h, the CzT-CMOP-1 was isolated by centrifuging and washed with water, methanol, and hexane. The FTIR spectrum of this isolated CzT-CMOP-1 observed similar FTIR bands of the spectrum appeared with the as-synthesized CzT-CMOP-1 (Figure S4). This finding suggested the high chemical stability of our carbazole- and Tröger’s base-derived covalent microporous organic polymer in KOH (6 M) electrolyte.

## 4. Conclusions

We have synthesized a novel covalent microporous organic polymer containing carbazole and Tröger’s base CzT-CMOP via the polymerization of 9-(4-aminophenyl)-carbazole-3,6-diamine (Cz-3NH_2_) with dimethoxymethane. According to spectroscopic analyses and electronic structure calculations, the regioselectivity of such polymerization indicated that CMOP-1 formed through the second and seventh positions of the carbazole unit is favorable than the other isomer CMOP-2 formed through the fourth and fifth positions of the carbazole. Based on electrochemical experiments, the polymer exhibited high electrochemical performance of 240 F g^−1^ at a current density of 0.5 A g^−1^ with excellent stability after 2000 cycles when used as an electrode in supercapacitor due to the presence of redox-active carbazole unit on its skeleton. Accordingly, the synthesis of such organic porous polymer containing redox moiety appears to be useful, and the obtained material could be used for the fabrication of extremely effective energy storage devices, in addition to other energy-related applications.

## Data Availability

The data presented in this study are available on request from the corresponding author.

## Supplementary Materials

The following are available online at https://www.mdpi.com/article/10.3390/polym13091385/s1, Scheme S1: Synthesis of the 3,6-dinitro-9-(4-nitrophenyl)-carbazole (Cz-3NO_2_). Figure S1: ^1^H-NMR spectrum of 9-(4-aminophenyl)-carbazole-3,6-diamine (Cz-3NH_2_). Figure S2: ^13^C-NMR spectrum of 9-(4-aminophenyl)-carbazole-3,6-diamine (Cz-3NH_2_). Figure S3: DSC thermogram of the CzT-CMOP-1. Figure S4: FTIR spectra of CzT-CMOP-1 as-synthesized and after 48 h treatment with 6 M KOH. Table S1: Comparison between the specific surface area and specific capacitance of the CzT-CMOP with those of previously reported organic polymers for supercapacitor application.
