# Supplementary material for: A Tröger’s Base-Derived Covalent Organic Polymer Containing Carbazole Units as a High-Performance Supercapacitor
Source: Polymers (Basel). 2021 Apr 24;13(9):1385. doi: 10.3390/polym13091385 (PMC8123202; doi:10.3390/polym13091385)
Supplement: Supplementary file 1 [file polymers-13-01385-s001.zip › polymers-1163383-supplementary.pdf]

Supporting Information

A Tröger's base-derived covalent organic polymer containing carbazole units as a high-performance supercapacitor

Ahmed F. M. EL-Mahdy [1,*], Johann Lüder [1], Mohammed G. Kotp[1] and Shiao-Wei Kuo[1]

[1]Department of Materials and Optoelectronic Science, National Sun Yat-Sen University, Kaohsiung, 80424, Taiwan

*To whom correspondence should be addressed

E-mail: **ahmedelmahdy@mail.nsysu.edu.tw**

Characterization

Proton and carbon nuclear magnetic resonance (^1H and ^{13}C NMR) spectra were recorded using an INOVA 500 instrument with DMSO-d_6 and $CDCl_3$ as solvents and tetramethylsilane (TMS) as the external standard. Chemical shifts are provided in parts per million (ppm). Fourier transform mass spectra (electrospray ionization, ESI) of were recorded using a Bruker Solarix spectrometer. FTIR spectra were recorded using a Bruker Tensor 27 FTIR spectrophotometer and the conventional KBr plate method; 32 scans were collected at a resolution of 4 cm^{-1}. Solid state nuclear magnetic resonance (SSNMR) spectra were recorded using a Bruker Avance 400 NMR spectrometer and a Bruker magic angle spinning (MAS) probe, running 32,000 scans. Cross-polarization with MAS (CPMAS) was used to acquire ^{13}C NMR spectral data at 75.5 MHz. The CP contact time was 2 ms; ^1H decoupling was applied during data acquisition. The decoupling frequency corresponded to 32 kHz. The MAS sample spinning rate was 10 kHz. Field emission scanning electron microscopy (FE-SEM) was conducted using a JEOL JSM-7610F scanning electron microscope. Samples were treated via Pt sputtering for 100 s prior to observation. Transmission electron microscope (TEM) images were obtained with a JEOL JEM-2010 instrument operated at 200 kV. BET surface area and porosimetry measurements of the prepared samples (ca. 20–100 mg) were performed using a Micromeritics ASAP 2020 Surface Area and Porosity analyzer. Nitrogen isotherms were generated through incremental exposure to ultrahigh-purity N_2 (up to ca. 1 atm) in a liquid nitrogen (77 K) bath. Surface parameters were determined using BET adsorption models in the instrument's software. TGA was performed using a TA Q-50 analyzer under a flow of N_2 atmosphere. The samples were sealed in a Pt cell and heated from 40 to 800 °C at a heating rate of 20 °C min^{-1} under a flow of N_2 atmosphere at a flow rate of 50 mL min^{-1}.

Synthetic Procedures

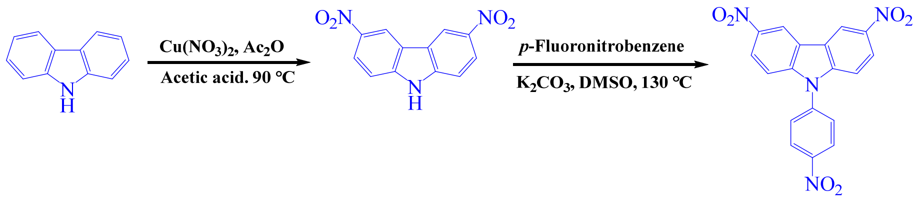

Carbazole = Cz   Cz-2NO_2 Cz-3NO_2

Scheme S1. Synthesis of the 3,6-dinitro-9-(4-nitrophenyl)-carbazole (Cz-3NO_2).

Synthesis of 3,6-dinitro-9*H*-carbazole (Cz-2NO_2)

In a 250 mL two neck-bottle, $Cu(NO_3)_2$•2.5H_2O (7.3 g, 30 mmol) was firstly dissolved in a mixture of acetic anhydride (30 mL) and acetic acid (20 mL) at room temperature. To this homogenous solution, carbazole (4.2 g, 25 mmol) was added in small portions over 15 min at 15-20 °C. After complete addition, the temperature of the reaction mixture was allowed to warm to room temperature over a period of 30 min and then to 90 °C for further 30 min. The mixture was poured into distilled water (250 mL) with constant stirring. The precipitate was collected by filtration, and washed five times with distilled water (100 mL). To separate 3,6-dinitro-9*H*-carbazole (Cz-2NO_2) from the formed isometric dinitrocarbazoles, the collected precipitated (2 g) was dissolved into 130 mL of alcoholic potassium hydroxide solution (60 g KOH into 1L of ethanol). After being stirred for 30 min at 50 °C, the insoluble solid was isolated by filtration and washed three times with distilled water (20 mL). The red alkaline alcoholic filtrate was acidified with concentrated hydrochloric acid to give a yellow solid product. The yellow precipitate was then collected by filtration, washed three times with distilled water (20 mL) and dried at 100 °C under vacuum. Chromatography on silica gel, eluting with petroleum ether/EtOAc (3:1) gave 3,6-dinitro-9*H*-carbazole (Cz-2NO_2) as yellow solid 5.16 g (85%). Mp: 244-245 °C. FT-IR (powder): 3400, 3091, 1611, 1583, 1519, 1484, 1339, 1310, 1245, 1098, 898, 812. ^{1}H NMR (DMSO-d_6, 25 °C, 500 MHz): δ = 12.69 (s, 1H), 9.48 (d, J = 3.0 Hz, 2H), 8.39 (dd, J = 9.0, 3.0 Hz, 2H), 7.76 (d, J = 9.0 Hz, 2H). ^{13}C NMR ((DMSO-d_6, 25 °C, 125 MHz): δ = 144.54, 141.09, 122.62, 122.38, 118.76, 112.29.

Synthesis of 3,6-dinitro-9-(4-nitrophenyl)-carbazole (Cz-3NO$_2$)

In a 100 mL two neck round-bottomed flask under a N_2 atmosphere flow, a solution of 3,6-dinitro-9*H*-carbazole (2 g, 7.77 mmol) and potassium carbonate (5.37 g, 38.85 mmol) in dry DMSO (40 mL) was stirred for 10 min. Then, 1-Fluoro-4-nitrobenzene (1.65 mL, 15.55 mmol) was added with stirring, and the reaction mixture was heated at 140 °C for 24 h. The reaction mixture was cooled to room temperature and poured into distilled water (100 mL) slowly to give a brown solid precipitate. The formed product was collected by filtration, washed thoroughly with distilled water (50 mL), and dried under vacuum to yield 3,6-dinitro-9-(4-nitrophenyl)-carbazole as a brown solid 2.35 g (80%). Mp: > 300 °C. FT-IR (powder): 3084, 1611, 1591, 1587, 1510, 1335, 1300, 1273, 1231, 1170, 1104, 854, 839. ^{1}H NMR (DMSO-d_6, 25 °C, 500 MHz): δ = 9.30 (s, 1H), 9.07 (s, 1H), 8.83 (d, J = 7.8 Hz, 1H), 8.49 (d, J = 4.8 Hz, 2H), 8.13 (d, J = 9.6 Hz, 1H), 7.93 (d, J = 7.8 Hz, 1H), 7.91(d, J = 9.6 Hz, 1H), 7.75(d, J = 4.8 Hz, 2H). ^{13}C NMR ((DMSO-d_6, 25 °C, 125 MHz): δ = 161.67, 144.94, 127.13, 120.48, 119.49, 117.21, 114.92, 112.64.

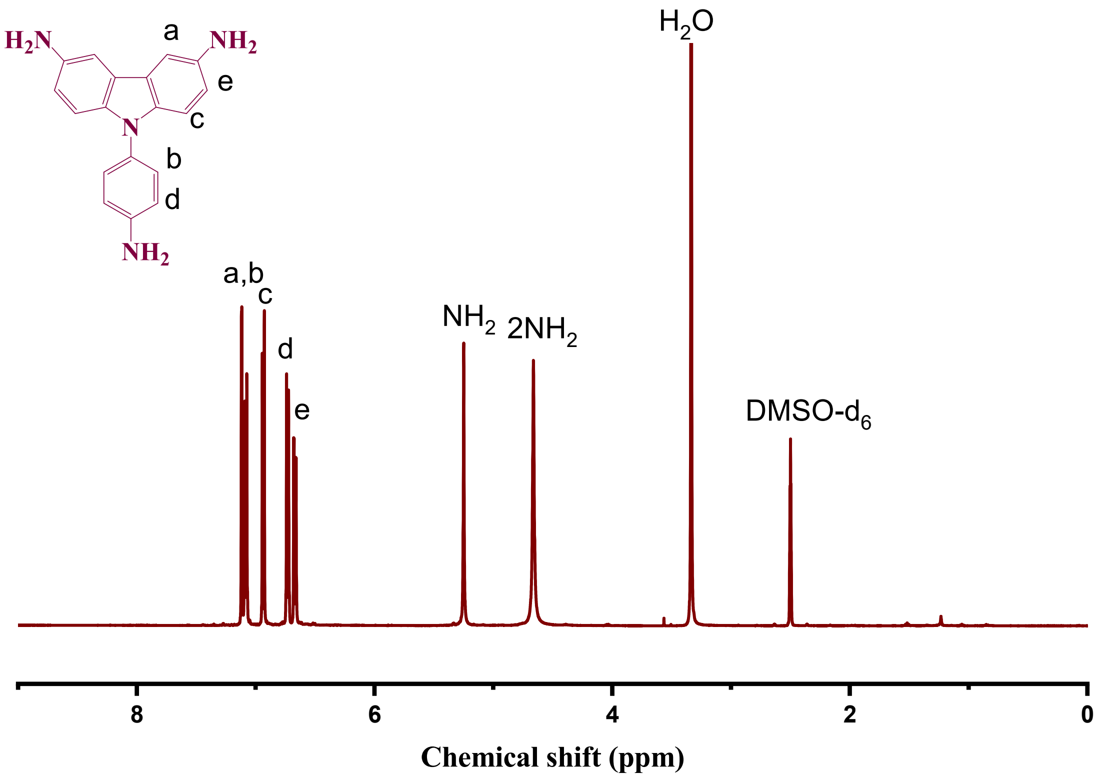

Figure S1. ^{1}H-NMR spectrum of 9-(4-aminophenyl)-carbazole-3,6-diamine (Cz-3NH$_2$).

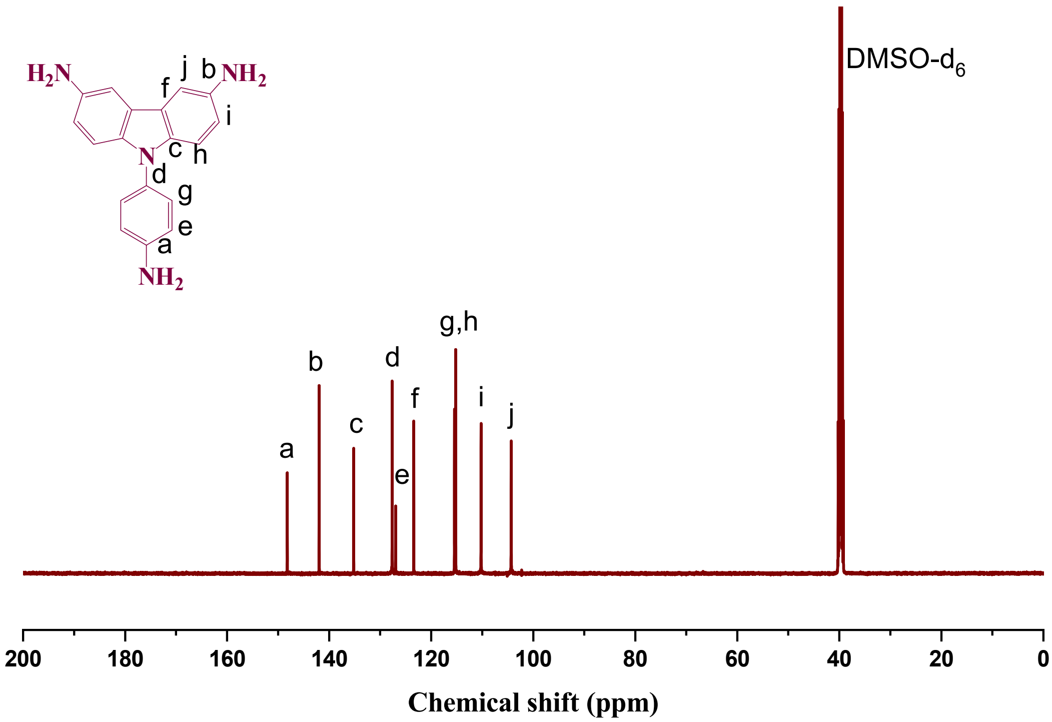

Figure S2. ^{13}C-NMR spectrum of 9-(4-aminophenyl)-carbazole-3,6-diamine (Cz-3NH2).

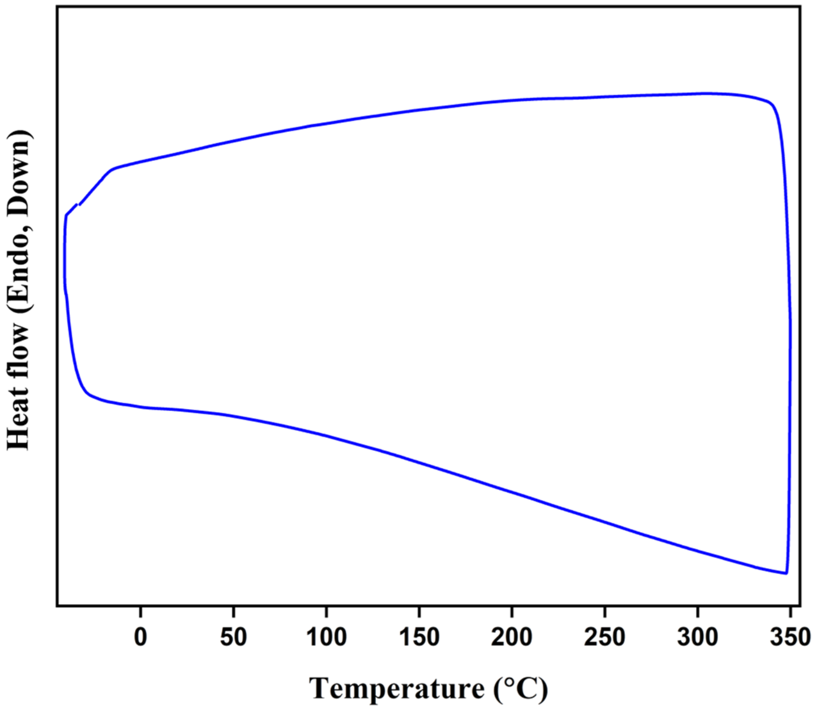

Figure S3. DSC thermogram of the CzT-CMOP-1.

Table S1. Comparison between the specific surface area and specific capacitance of the CzT-CMOP with those of previously reported organic polymers for supercapacitor application.

Materials	S_{BET} (m^2 g^{-1})	Capacitance	Ref.
Car-TPA COF	1334	13.6 F g^{-1} at 0.2 A g^{-1}	S1
Car-TPP COF	743	14.5 F g^{-1} at 0.2 A g^{-1}	S1
Car-TPT COF	721	17.4 F g^{-1} at 0.2 A g^{-1}	S1
DAAQ-TFP COF	1280	48 ± 10 F g^{-1} at 0.1 A g^{-1}	S2
TPA-COF-1	714	51.3 F g^{-1} at 0.2 A g^{-1}	S3
TPA-COF-2	478	14.4 F g^{-1} at 0.2 A g^{-1}	S3
TPA-COF-3	557	5.1 F g^{-1} at 0.2 A g^{-1}	S3
TPT-COF-4	1132	2.4 F g^{-1} at 0.2 A g^{-1}	S3
TPT-COF-5	1747	0.34 F g^{-1} at 0.2 A g^{-1}	S3
TPT-COF-6	1535	0.24 F g^{-1} at 0.2 A g^{-1}	S3
HOMCNSs	502	72.79 F g^{-1} at 0.5 A g^{-1}	S4
An-CPOP-1	700	72.75 F g^{-1} at 0.5 A g^{-1}	S5
An-CPOP-2	700	98.40 F g^{-1} at 0.5 A g^{-1}	S5
TPE-HPP	922	67.00 F g^{-1} at 0.5 A g^{-1}	S6
DPT-HPP	1230	110.5 F g^{-1} at 0.5 A g^{-1}	S6
Fc-CMPs	653.2	147 F g^{-1} at 0.5 A g^{-1}	S7
CMPs	672.3	72 F g^{-1} at 0.5 A g^{-1}	S7
GH-CMP	219	206 F g^{-1} at 0.5 A g^{-1}	S8
**CzT-CMOP-1**	**615**	**240 F g^{-1} at 0.5 A g^{-1}**	**This work**

Stability of CzT-CMOP-1 in 6M KOH

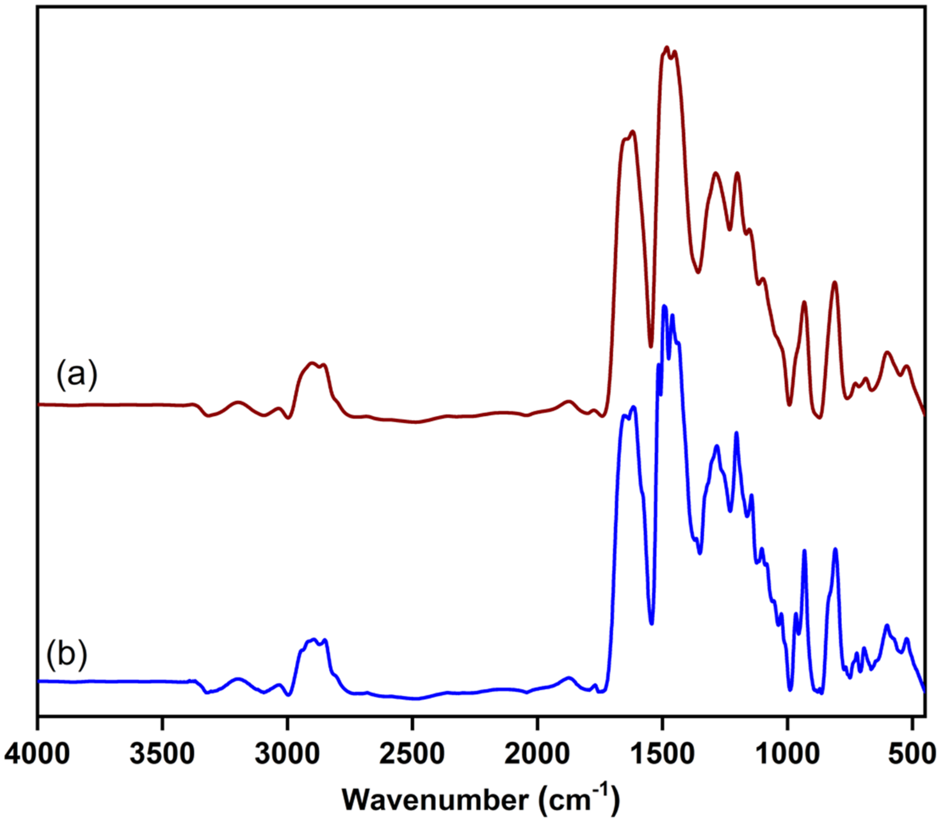

Figure S4. FTIR spectra of CzT-CMOP-1 as-synthesized and after 48 h treatment with 6 M KOH.

References

S1. EL-Mahdy, A.F.M.; Young, C.; Kim, J.; You, J.; Yamauchi, Y.; Kuo, S.W.; Hollow Microspherical and Microtubular [3 + 3] Carbazole-Based Covalent Organic Frameworks and Their Gas and Energy Storage Applications. *ACS Appl. Mater. Interfaces* **2019**, *11*, 9343-9354.

S2. DeBlase, C.R.; Silberstein, K.E.; Truong, T.T.; Abruña, H.D.; Dichtel, W.R.; β-KetoenamineLinked Covalent Organic Frameworks Capable of Pseudocapacitive Energy Storage. *J. Am. Chem. Soc.* **2013**, *135*, 16821-16824.

S3. El-Mahdy, A.F.M.; Kuo, C.H.; Alshehri, A.A.; Kim, J.; Young, C.; Yamauchi, Y.; Kuo, S.W.; Strategic Design of Triphenylamine- and Triphenyltriazine-Based Two-Dimensional Covalent Organic Frameworks for CO_2 Uptake and Energy Storage. *J. Mater. Chem. A* **2018**, *6*, 19532-19541.

S4. Ping, J.; Zhang, W.; Yu, P.; Pang, H.; Zheng, M.; Dong, H.; Hu, H.; Xiao, Y.; Liu, Y.; Liang, Y.; Improved Ion-diffusion Performance by Engineering an Ordered Mesoporous Shell in Hollow Carbon Nanospheres. *Chem. Commun.* **2020**, *56*, 2467-2470.

S5. Mohamed, M.G.; X. Zhang, X.; Mansoure, T.H.; EL-Mahdy, A.F.M.; Huang, C.F.; Danko, M.; Xin, Z.; Kuo, S.W. Hypercrosslinked Porous Organic Polymers Based On Tetraphenylanthraquinone for CO_2 Uptake and High-Performance Supercapacitor. *Polymer* **2020**, *205*, 122857.

S6. Mohamed, G.M.; EL-Mahdy A.F.M.; Meng, T.-S.; Samy, M.M.; Kuo, S.W. Multifunctional Hypercrosslinked Porous Organic Polymers Based on Tetraphenylethene and Triphenylamine Derivatives for High-Performance Dye Adsorption and Supercapacitor. *Polymers* **2020**, *12*, 2426.

S7. Khattak, A.M.; Sin, H.; Ghazi, Z.A.; He, X.; Liang, B.; Khan, N.A.; Alanagh, H.R.; Iqbal, A.; Li, L; Tang, Z. Controllable Fabrication of Redox-Active Conjugated Microporous Polymers On Reduced Graphene Oxide for High Performance Faradaic Energy Storage. *J. Mater. Chem. A,* **2018**, *6*, 18827.

S8. Zhang, M.; Zhao, T.; Dou, J.; Xu, Z.; Zhang, W.; Chen, X.; Wang, X.; Zhou, B. Bottom-Up Construction of Conjugated Microporous Polyporphyrin-Coated Graphene Hydrogel Composites with Hierarchical Pores for High-Performance Capacitors. *ChemElectroChem,* 2019, **6**, 5946-5950.
